# Lactose Intolerance: Lack of Evidence for Short Stature or Vitamin D Deficiency in Prepubertal Children 

**DOI:** 10.1371/journal.pone.0078653

**Published:** 2013-10-25

**Authors:** Nithya Setty-Shah, Louise Maranda, Ninfa Candela, Jay Fong, Idris Dahod, Alan D. Rogol, Benjamin Udoka Nwosu

**Affiliations:** 1 Department of Pediatrics, University of Massachusetts Medical School, Worcester, Massachusetts, United States of America; 2 Department of Quantitative Health Sciences, University of Massachusetts Medical School, Worcester, Massachusetts, United States of America; 3 Department of Pediatrics, Saint Vincent Hospital, Worcester, Massachusetts, United States of America; 4 Department of Pediatrics, University of Virginia Health System, Charlottesville, Virginia, United States of America; Indiana University, United States of America

## Abstract

**Background:**

The health consequences of lactose intolerance (LI) are unclear.

**Aims:**

To investigate the effects of LI on stature and vitamin D status.

**Hypotheses:**

LI subjects will have similar heights and vitamin D status as controls.

**Subjects and Methods:**

Prepubertal children of ages 3-12 years with LI (n=38, age 8.61 ± 3.08y, male/female 19/19) were compared to healthy, age- and gender-matched controls (n=49, age 7.95±2.64, male/female 28/21). Inclusion criteria: prepubertal status (boys: testicular volume <3cc; girls: Tanner 1 breasts), diagnosis of LI by hydrogen breath test, and no history of calcium or vitamin D supplementation. Vitamin D deficiency was defined as 25-hydroxyvitamin D [25(OH)D] <50 nmol/L. Gender-adjusted midparental target height (MPTH) z-score was calculated using NCHS data for 18 year-old adults. Data were expressed as mean ± SD.

**Results:**

There was no significant difference in 25(OH)D between the LI and non-LI subjects (60.1±21.1, vs. 65.4 ± 26.1 nmol/L, *p* = 0.29). Upon stratification into normal weight (BMI <85^th^ percentile) vs. overweight/obese (BMI ≥85^th^ percentile), the normal weight controls had significantly higher 25(OH)D level than both the normal weight LI children (78.3 ± 32.6 vs. 62.9 ± 23.2, p = 0.025), and the overweight/obese LI children (78.3±32.6 vs. 55.3±16.5, *p* = 0.004). Secondly, there was no overall difference in height z-score between the LI children and controls. The normal weight LI patients had similar height as normal controls (-0.46 ± 0.89 vs. -0.71 ± 1.67, *p* = 0.53), while the overweight/obese LI group was taller than the normal weight controls (0.36 ± 1.41 vs. -0.71 ± 1.67, *p* = 0.049), and of similar height as the overweight/obese controls (0.36 ± 1.41 vs. 0.87 ± 1.45, *p* = 0.28). MPTH z-score was similar between the groups.

**Conclusion:**

Short stature and vitamin D deficiency are not features of LI in prepubertal children.

## Introduction

There is no consensus on the health consequences of lactose intolerance (LI)[1,2]. The commonest form of LI, primary LI, results from either a genetic inability to produce lactase, an enzyme that breaks down lactose to glucose and galactose, or from an age-related down-regulation of lactase production[3]. Bacterial fermentation of the non-digested lactose leads to the production of short chain fatty acids and gas including hydrogen, carbon dioxide, and methane[4]. The degree of elevation of these gases as measured by the hydrogen breath test forms the basis of the clinical diagnosis of LI[4]. The associated bloating, diarrhea, and abdominal pain lead to the avoidance of dairy products[5]. A central hypothesis in LI is that dairy avoidance leads to reduced intake of protein and calcium, which consequently results in poor growth, short stature and low bone mineral density, respectively [6]. However, no systematic study has been conducted in prepubertal children to accurately assess this hypothesis. 

Even though the studies that demonstrated an association between LI and low bone mineral density have provided important information in this field [6-8], they failed to articulate a coherent biochemical basis for the reported low bone mineral density and increased fracture risk in their cohorts as they provided no data on the vitamin D status of these lactose intolerant children and their control groups. It is unclear whether the bone findings from the above referenced studies were due to insufficient calcium intake alone, or whether there was an added effect from vitamin D deficiency as a result of reduced intake of vitamin D-fortified dairy products. This is crucial because calcium absorption in the gastrointestinal tract depends on vitamin D-mediated processes. Therefore, a coherent hypothesis on low bone mineral density in LI should take into consideration the primary role of vitamin D in calcium absorption. Our research question was whether prepubertal children with LI had significantly lower vitamin D levels compared to their age and gender-matched peers. 

Another controversial area in LI research is whether this condition is associated with short stature. Although some investigators have suggested that adequate calcium intake during the growth period may be critical for reaching optimal bone growth during the growing years[9], others have provided evidence of short stature in children with LI, milk allergy, or those on milk-elimination diets[10-12]. Further studies in prepubertal children showed that long term avoidance of cow’s milk was associated with small stature and diminished bone health[6,10]. Even though several studies have shown positive associations between milk intake and height in prepubertal children[13-16], others have shown no such relationships[17,18]. A recent analysis reports that the biological sequalae of routine milk consumption, such as statural growth, are not well understood[19]. Even though the circulating levels of the growth promoting peptide, insulin-like growth factor-I (IGF-I), rises after milk consumption[20], it is unclear whether the rise in IGF-I levels is due to the IGF-I contained in milk or as a result of endogenous IGF-I production[20]. More importantly, because elevated serum IGF-I levels limit growth hormone production via a negative feedback mechanism[21,22], the net effect of milk-associated IGF-I elevation on stature in prepubertal children is unclear.

Because of the lack of a well-designed study on the effect of LI on stature and vitamin D metabolism, gastroenterologists are unable to provide the much needed prognostic information on bone health and stature to parents and guardians of children with newly-diagnosed LI. 

This study was designed to address these two important questions and provide the needed data on height and vitamin D status of these children. The primary aim of this study was to compare the vitamin D status of prepubertal children with LI to non-affected children of similar age, gender, and prepubertal status. The study’s primary hypothesis was that children with LI would have similar vitamin D status as their healthy peers. The second aim of the study was to investigate whether prepubertal children with LI were significantly shorter than their healthy peers. The study’s secondary hypothesis was that children with LI would be of similar height as their healthy peers.

To test these hypotheses, a two-site cross-sectional study was conducted in prepubertal children with a diagnosis of LI who also had a 25(OH)D level measured. These results were compared to a cohort of healthy prepubertal children who participated in a prospective cross-sectional study on the role of vitamin D metabolism on bone mineral content (clinical trial identifier NCT00756899).

## Subjects and Methods

The study protocol was approved by the Institutional Review Boards of the University of Massachusetts and the Saint Vincent Hospital, Worcester, Massachusetts. The control group (n=49) was drawn from a study to evaluate the role of vitamin D status on bone mineral density. Its clinical trial identification number is NCT00756899. Written informed consent was obtained from each subject’s parent or legal guardian and assent was obtained from each subject prior to participating in the study. The study group (n=38) consisted of prepubertal children of ages 3-12 years who were diagnosed with LI at the Saint Vincent Hospital and the UMassMemorial Medical Center between 2008 and 2012. 

### Subjects

The control group consisted of healthy children who were recruited by means of advertisement using paper flyers distributed to the primary care physician offices in Central New England, USA. Fifty subjects signed consent for the study. Forty-nine subjects (28 males and 21 females) between 3-12 years of age were studied. The mean age of the cohort was 7.95 ± 2.63 years; mean age of females 7.09 ± 2.37; mean age of males 8.59 ± 2.68 years. All participants were of prepubertal status: males, with testicular volume of ≤ 3 cc, and females with Tanner Stage 1 breasts as determined by palpation by the principal investigator. Subjects were excluded if they had any known metabolic or genetic diseases resulting in obesity such as severe hypothyroidism, pseudohypoparathyroidism, or Cushing’s syndrome. We also excluded patients with systemic illnesses such as diabetes mellitus, nephrotic syndrome, chronic renal disease, or chronic inflammatory disorders. Subjects with a history of significant weight loss or gain (change of >10% body weight in 6 months) were excluded from the study. Methods used for exclusion included history, physical examination, and screening laboratory tests for fasting blood glucose, cortisol, urinalysis, comprehensive metabolic panel, serum creatinine, and thyroid function tests. 

Subjects were included in the study group if they had a diagnosis of LI confirmed by hydrogen breath test. All study subjects were prepubertal, and had no history of secondary causes of LI such as celiac disease, infectious enteritis, and Crohn’s disease. A further inclusion criterion was the availability of 25(OH)D level measured at the time diagnosis of LI. The estimation of 25(OH)D at the time of diagnosis is a routine practice at both centers. Patients were excluded if they had diseases of calcium metabolism or vitamin D metabolism, and were receiving calcium, vitamin D or multivitamin supplementation. Seven eligible patients were excluded because they were receiving vitamin D supplements prior to the hydrogen breath test.

### Study Methods

Participants in both the study group and control group were evaluated between 0800-1100 hours following an overnight fast. 

#### Anthropometry

Height was measured to the nearest 0.1 cm using a wall-mounted stadiometer (Holtain Ltd, Crymych, Dyfed, UK) that was calibrated daily. Weight was measured to the nearest 0.1 kg using an upright scale. BMI was derived using the formula weight/height^2^ (kg/m^2^), and expressed as z-score for age and gender based on National Center for Health Statistics (NCHS) data [23]. Gender-adjusted mid-parental target height (MPTH) z-score was calculated for 18 year old adults using NCHS data and the standard formula for MPTH[24]. The MPTH is a child's projected adult height based on the heights of his or her parents and is calculated as follows: for girls, the father's height minus 13 cm (5 in) is averaged with the mother's height; for boys, the mother's height plus 13 cm is averaged with the father's height[24]. Anthropometric data were expressed as mean ± SD. Heights of parents were obtained by history[25] and some by measurement in the clinic. Bone mineral density was not measured because of funding constraints.

#### Hydrogen Breath Test: Lactest by Quintron[26] (orange-flavored lactose)

Prior to the testing, all patients were instructed not to take any form of antibiotics 10 days prior to the day of testing; not to ingest a high fiber diet one week prior to the test; and not to smoke, brush their teeth, or take any medications on the morning of the test; and to fast overnight for the test. All patients ingested Lactose at a dose of 1 g/kg body weight up to 25 grams dissolved in 8 ounces of water. Breaths were sampled at baseline 0, 30, 60, 90, and 120 minutes or until hydrogen/methane (H_2_/CH_4_) ppm reached 20/12 over the lowest preceding measures. Positive results were defined by the following criteria: (a) breath H_2_ level increase of at least 20 ppm over the lowest preceding value within the test period; (b) breath CH_4_ level increase of at least 12 ppm over the baseline within the test period; (c) combined increase of at least 15 ppm within the test period. 

#### Biochemical Study

A single venous blood sample was collected for serum 25(OH)D estimation between 0800 and 0900 hours. 

#### Assay

Serum levels of 25(OH)D were analyzed using 25-hydroxy chemiluminescent immunoassay (DiaSorin Liaison; Stillwater, Minnesota), which has a 100% cross-reactivity with both metabolites of 25(OH)D namely, 25(OH)D_2_ and 25(OH)D_3_ and thus measures total serum 25(OH)D content. Its functional sensitivity is 10 nmol/L (4 ng/mL), and its intra- and inter-assay coefficients of variation are 5% and 8.2% respectively. Vitamin D status was classified according to American Academy of Pediatrics and the Institutes of Medicine criteria as deficient, 25(OH)D <50 nmol/L (<20 ng/mL); or sufficient, 25(OH)D >50 nmol/L (>20 ng/mL)[27]. Because vitamin D status could vary with sunlight exposure and the seasons, we categorized each subject’s visit according to the seasons as follows: fall (September 22 – December 21), winter (December 22- March 21), spring (March 22 – June 21), and summer (June 22-September 21)[28].

## Statistical Analyses

Statistical analyses were performed using SPSS v. 21 (IBM Corporation, Somers, NY). 

Normal probability plots were constructed with the variables of interest. No departure from normality was detected, allowing for the use of parametric tests, without the need for transformation. Means, standard deviations and percentages were calculated for descriptive summary statistics. Proportions were compared using Fisher’s exact test. To adjust for the influence of adiposity on both height and 25(OH)D levels, subjects were stratified into normal weight vs. overweight/obese groups. The rationale for this stratification is because obesity is associated with vitamin D deficiency, as vitamin D is subject to either sequestration[29] or volumetric dilution[30] in fat depots. Equally, overweight/obese children are generally taller than their normal weight peers[31,32]. Overweight was defined as BMI of ≥85^th^ but <95^th^ percentile, while obesity was defined as a BMI of > 95^th^ percentile for age and sex. Differences between the four subgroups (normal weight vs. overweight/obese controls and study subjects) were first explored using a two-way ANOVA [F (3,86) = 4.731, p = 0.004], followed by post-hoc comparisons. Data were expressed as mean ± standard deviation (SD). 

## Results

Table 1 shows the mean (±SD) values for age, gender, seasonality, anthropometric, and biochemical parameters of the LI subjects and normal controls. The control subjects had significantly higher weight and BMI z-score compared to the LI children. There were no significant differences in height z-score, MPTH z-score, or 25(OH)D level between the subjects and controls.

**Table 1:** Comparative Analysis of the Characteristics of Children with Lactose Intolerance and Controls.

Parameters	Lactose Intolerant n=38	Controls n=49	*p*
Age (years)	8.62 ± 3.08	7.95 ± 2.63	0.29
Sex (% males)	50.00	57.14	0.51
Race (% white)	73.7	85.7	0.18
Height SDS	-0.16 ± 1.16	0.19 ± 1.71	0.27
Weight SDS	0.32 ± 1.38	1.05 ± 1.97	0.045
BMI SDS	0.56 ± 1.37	1.26 ± 1.62	0.031
MPTH SDS	0.0014 ± 1.31 (n=14)	0.025 ± 0.95 (n=26)	0.10
25(OH)D (nmol/L)	60.1 ± 21.1	65.4 ± 26.1	0.29
Season (% Summer-Fall)	47.37	34.69	0.23

SDS = standard deviation score; MPTH = mid-parental target height; 25(OH)D= 25-hydroxyvitamin D; BMI = body mass index

### 25: hydroxyvitmin D

**Figure 1:**
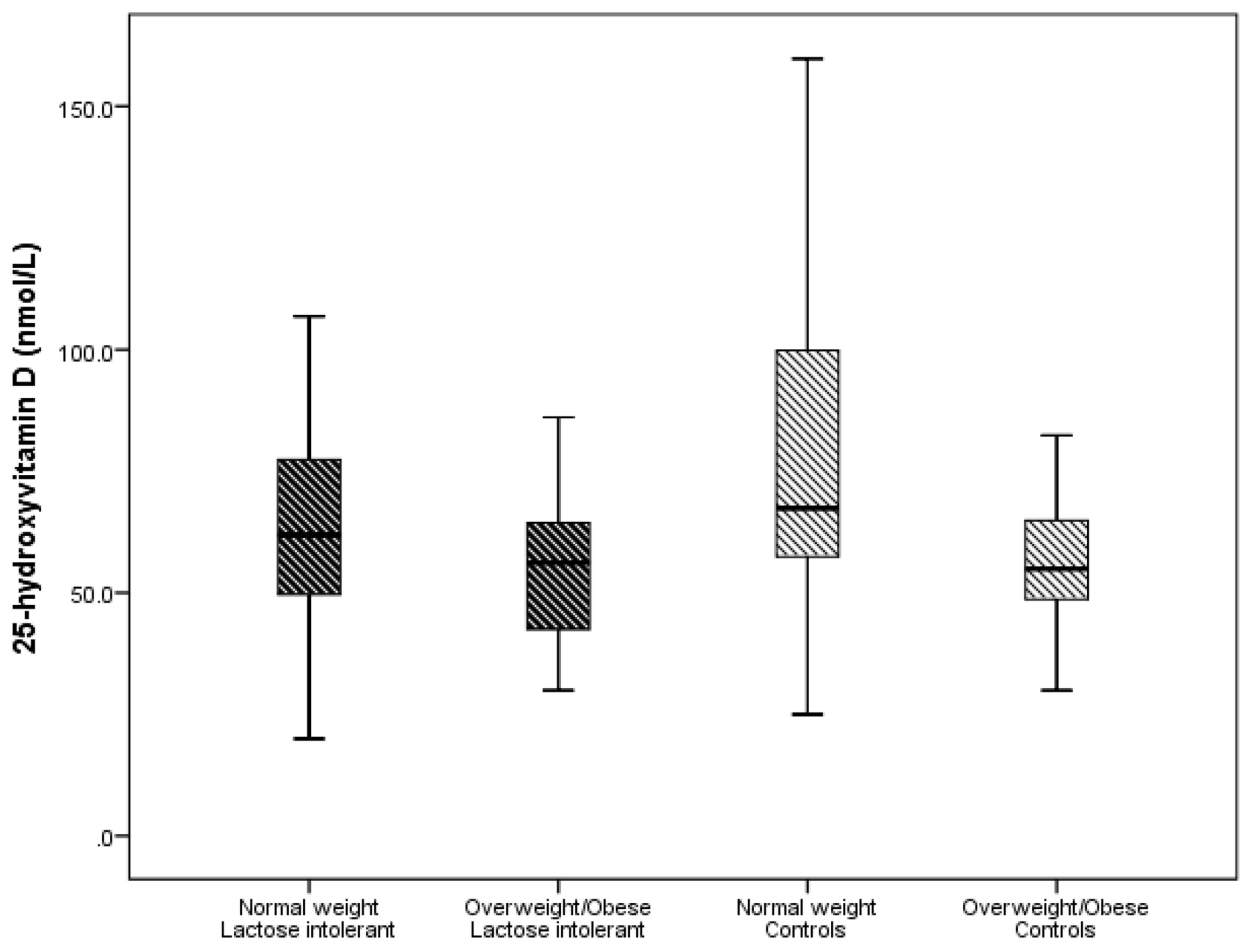
Box plots of the comparison of 25-hydroxyvitamin D values of patients with lactose intolerance and normal controls stratified by body mass index. This figure shows that the overweight/obese controls and overweight/obese lactose intolerant children had lower vitamin D levels compared to their respective normal weight peers. Note: 50 nmol/L=20 ng/mL.

The normal weight controls had significantly higher 25(OH)D levels than the normal weight LI children (78.3 ± 32.6 vs. 62.9 ± 23.2 nmol/L, p=0.025), and much greater levels than the overweight/obese LI children (78.3 ± 32.6 vs. 55.3 ± 16.5 nmol/L, p =0.004) (Figure 1). There was neither a difference in 25(OH)D level between the overweight/obese control subjects and overweight obese LI children (55.7 ± 13.9 vs. 55.3 ± 16.5 nmol/L, p=0.95), nor between the overweight/obese controls and normal weight LI children (55.7 ± 14.0 vs. 62.9 ± 23.2 nmol/L, p=0.26). Even though there was a significant difference between normal weight control and overweight/obese controls (78.3 ± 32.6 vs. 55.7 ± 13.9 nmol/L, p=0.001), there was no difference in 25(OH)D level between the normal weight LI and overweight/obese LI (62.9 ± 23.2 vs. 55.3 ± 16.5 nmol/L, p=0.32). 

An analysis of the racial/ethnic composition of the LI subjects and controls showed that both the LI subjects and controls were predominantly white. There were no significant differences in height and 25(OH)D levels among the racial/ethnic groups (Table 2). Equally, there were no significant differences in serum 25(OH)D levels across the seasons when the racial/ethnic groups were analyzed individually (Table 3). 

**Table 2:** Comparative Analysis of the Racial/Ethnic Composition of the Participants with respect to Height z-score and 25-hydroxyitamin D levels.

**Parameters**	**Lactose Intolerant**				**Controls**			
	Whites	Blacks	*Others	*p*	Whites	Blacks	*Others	*p*
N	29 (76.3%)	6 (15.8%)	3 (7.9%)	0.72	45 (91.8%)	2 (4.1%)	2 (4.1%)	0.50
Height z-score	-0.12 ±1.16	-0.05 ±1.47	-0.69 ± 0.63	0.71	0.21 ± 1.72	1.08 ± 0.12	-1.17 ± 2.55	0.42
25(OH)D (nmol/L)	63.2 ± 21.1	48.9 ± 13.7	51.9 ± 30.2	0.26	66.7 ± 26.8	46.2 ± 8.8	56.2 ± 1.8	0.50

*Others: Asians, Multi-racial, Pacific Islanders; 25(OH)D 25-hydroxyvitamin D

**Table 3:** A Comparative Analysis of the 25-hydroxyvitamin D levels in nmol/L Stratified by Race and Season.

**Season**										
	**Lactose intolerant**					**Controls**				
**Race**	**Summer**	**Fall**	**Winter**	**Spring**	***p***	**Summer**	**Fall**	**Winter**	**Spring**	***p***
**White**	n = 6	n=8	n=10	n=5		n=9	n=8	n=10	n=18	
**25(OH**)**D (nmol/L**)	72.1 ± 18.8	70.9 ± 20.0	52.9 ± 21.8	60.8 ± 20.2	0.21	84.03 ± 27.0	62.1 ± 18.3	53.9 ± 15.7	67.1 ± 31.3	0.09
**Black**	n=0	n=2	n=3	n=1		n=0	n=0	n=0	n=2	-
**25(OH**)**D (nmol/L**)	-	63.3 ± 1.6	38.9 ± 10.6	50.4	0.12	-	-	-	46.2 ± 8.8	
*Others	n=1	n=0	n=2	n=0		n=0	n=0	n=0	n=2	-
**25(OH**)**D (nmol/L**)	78.9		37.9 ± 25.4	-	0.41	-	-	-	56.2 ± 1.8	-

*Others: Asians, Multi-racial, Pacific Islanders; 25(OH)D 25-hydroxyvitamin D

### Height z-score

**Figure 2:**
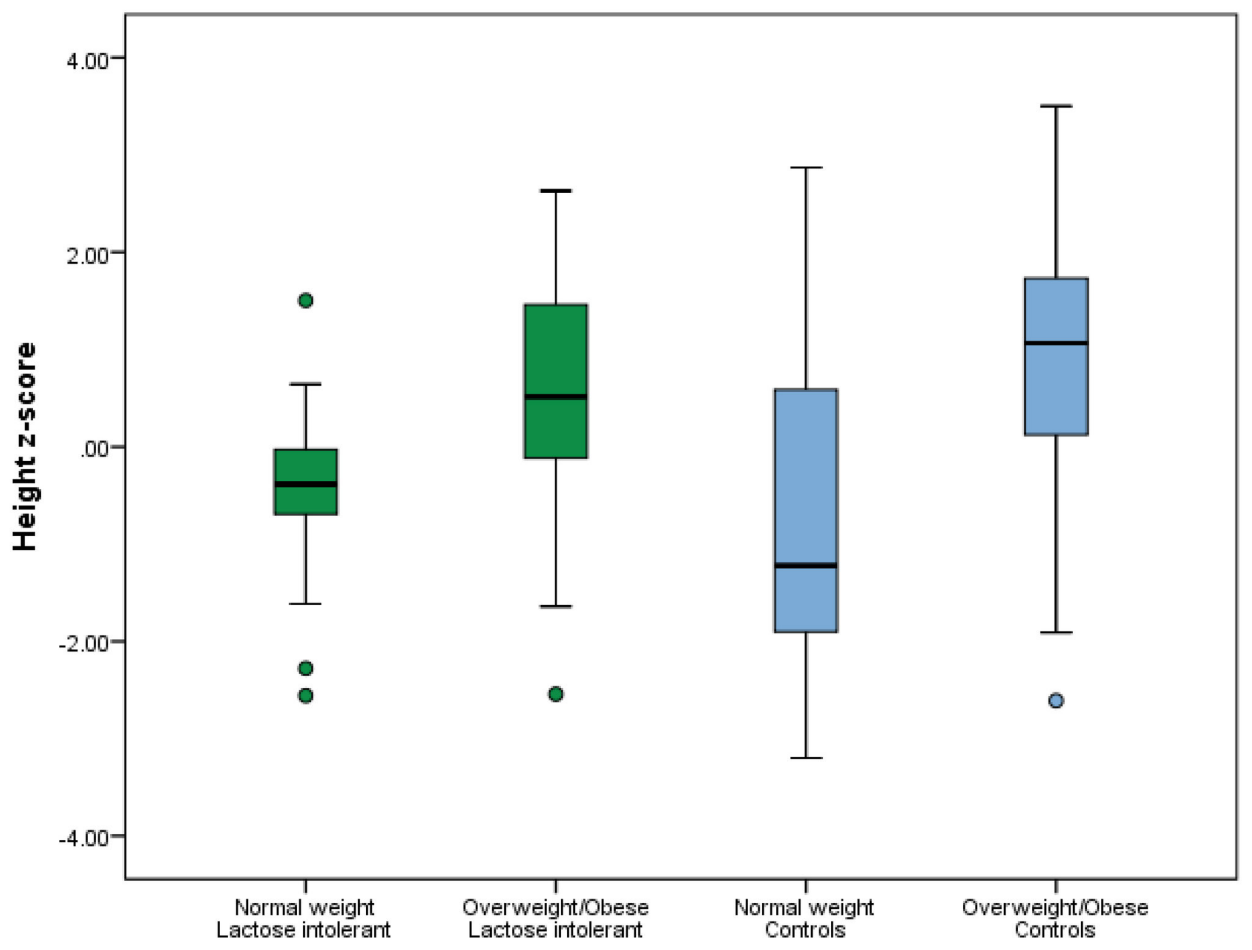
Box plots of the comparison of height standard deviation scores of patients with lactose intolerance and normal controls stratified by body mass index. This figure shows that the overweight/obese children with lactose intolerance were taller than the normal weight controls but of similar height as the overweight/obese controls.

Although there were no differences in height z-score nor MPTH z-score when all the LI children were compared to the normal controls (Table 1), there were differences when the two groups were subdivided into normal weight (BMI <85^th^ percentile) vs. overweight/obese (BMI ≥85^th^ percentile) (Figure 2).The normal weight LI patients were of similar height as normal weight controls (-0.46 ± 0.89 vs. -0.71 ± 1.67, p = 0.53), but were significantly shorter than the overweight/ obese controls (-0.46 ± 0.89 vs. 0.90 ± 1.45, p <0.001), and nearly so for the obese LI patients (-0.46 ± 0.89 vs. 0.36 ± 1.41, p = 0.067). Interestingly, the overweight/obese LI group was taller than the normal weight controls (0.36 ± 1.41 vs. -0.71 ± 1.67, p = 0.049), and of similar height as the overweight/obese controls (0.36 ± 1.41 vs. 0.90 ± 1.45, p = 0.28).

**Figure 3:**
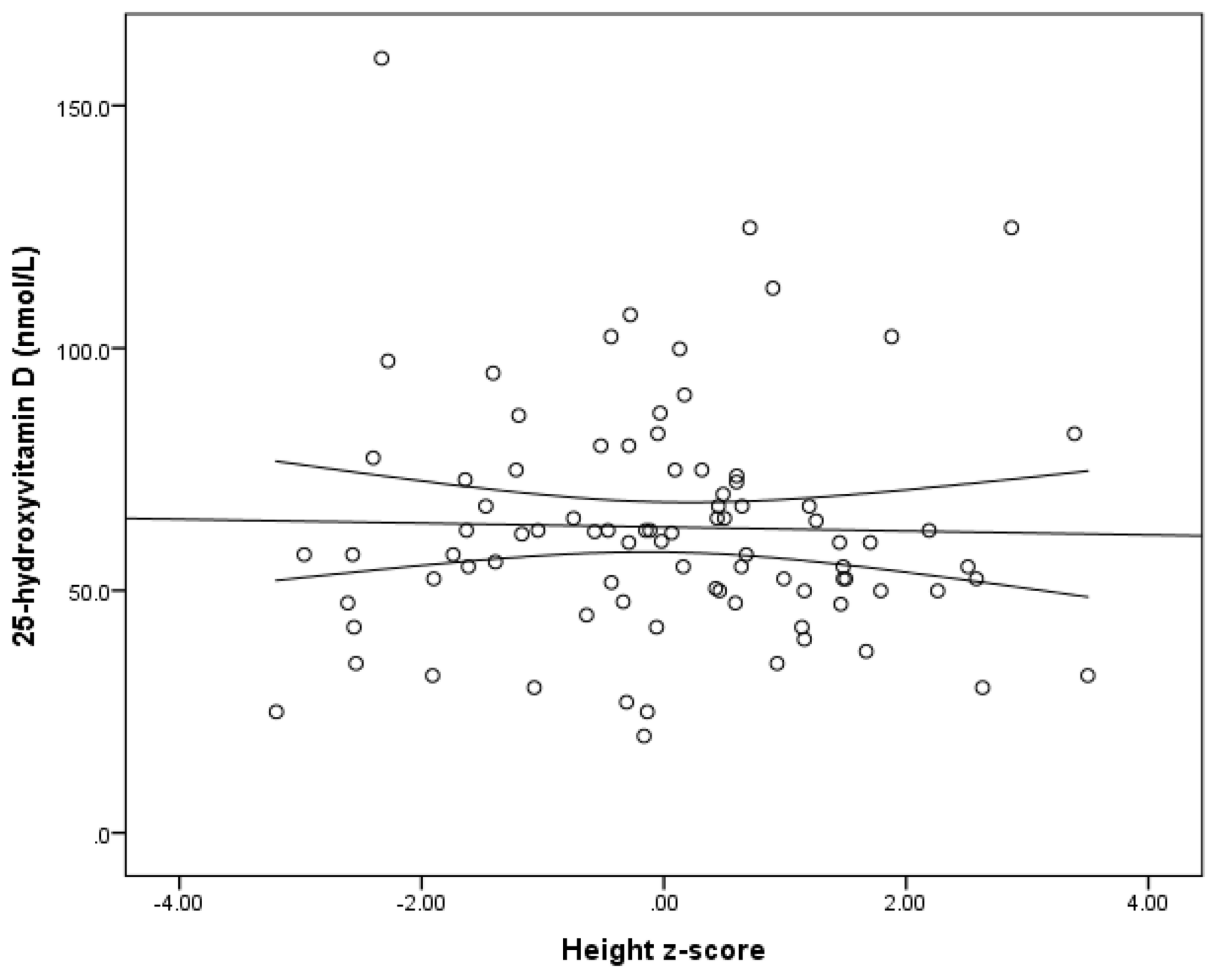
The relationship between height z-score and serum 25-hydroxyvitamin D concentration. This figure shows no relationship between height z-score and serum concentration of 25(OH)D in all subjects after adjusting for age, race, season, and BMI z-score (β=0.070, p = 0.562, R^2^=0.139).

There was no statistically significant relationship between height z-score and serum concentration of 25(OH)D in all subjects after adjusting for age, race, season, and BMI z-score (β = 0.070, p = 0.562, R^2^ = 0.139) (Figure 3).

## Discussion

This study showed no significant difference in the serum concentration of 25(OH)D between children with LI and their normal controls. However, when adjusted for BMI, both the normal weight LI- and overweight/obese LI patients had similar serum concentrations of 25(OH)D to the overweight/obese controls, but significantly lower 25(OH)D levels than the normal weight controls. Furthermore, children with LI had significantly reduced values for weight and BMI z-score, but no difference in height z-score or MPTH z-score compared to controls. There was no relationship between height z-score and serum concentration of 25(OH)D in all subjects. 

The findings from this study are important when viewed in light of the recent reports on LI from the Agency for Healthcare Research and Quality, and the National Institutes of Health Consensus statement which conclude that the evidence regarding the effect of dairy exclusion diets on long term gastrointestinal and bone health outcomes is relatively sparse in quantity and of low quality[1,2]. Available data do not strongly indicate that dairy-free diets are independently associated with poor long-term bone health outcomes[2]. This study’s finding of normal vitamin D status in these subjects may partially explain the absence of diminished long-term bone health outcomes in the reports cited above.

Though some studies have reported an association between calcium intake and bone mineral content (BMC) and BMD in children with LI or cow’s milk allergy[6,8,33], other investigators found no such associations[10,34] in carefully-designed studies that matched the experimental groups’ intake of calcium, milk-derived calcium, milk, cheese, yogurt, ice cream, and calcium density of the diet. The studies that reported associations between calcium intake and BMD did not evaluate the subjects’ vitamin D status[6,8,33]. Therefore, it is unclear whether the low BMD described in these studies was due to hypocalcemia secondary to poor calcium intake, or poor calcium absorption as a result of vitamin D deficiency. 

### 25: hydroxyvitamin D

This current study did not detect vitamin D deficiency, marked by serum 25(OH)D of <50 nmol/L, in our cohort with LI. This is not surprising as oral intake is not the only source of vitamin D, as this prohormone is synthesized in the skin through exposure to ultra-violet radiation. Hence, dietary intake of vitamin D is not necessarily required to maintain normal vitamin D status in individuals who maintain adequate exposure to sunlight. Vitamin D is also found in some foods, such as fish oil, egg yolk, fortified margarine, and breakfast cereals[35] which are available to most patients with LI.

We, however, showed that overweight/obese children had lower serum concentrations of 25(OH)D compared to the normal weight peers. This is consistent with other reports showing that increased adiposity is associated with vitamin D deficiency [36]. The mechanism of this association is unclear, however, proposed causative factors include: poor nutrition, inadequate exposure to sunlight, and the sequestration[29] and or volumetric dilution[30] of vitamin D in fat stores in overweight/obese individuals. Therefore, it is possible that overweight/obese subjects with LI may require a higher dose of vitamin D to maintain normal serum concentrations of 25(OH)D as the fat soluble, non-hydroxylated vitamin D is mainly stored in adipose tissue leading to reduced bioavailability[37].

### Height z-score

We did not detect a significant difference in height z-score between LI children and their unaffected peers in the initial analysis. However, when the subjects were stratified into normal weight vs. overweight/obese, the LI patients with BMI >85^th^ percentile were taller than the normal weight controls (BMI <85^th^ percentile), and nearly significantly taller than the normal weight LI subjects. The overweight/obese controls were not significantly taller than the overweight/obese LI subjects, but were significantly taller than the normal weight LI subjects.

Similar associations between height and adiposity in children have been reported in several regions of the world in studies carried out in the United States[31,38,39], Australia[32], and Japan[40]. The reason for the differential tall stature in overweight/obese prepubertal children is not fully known, but suggested mechanisms include: insulin stimulation of both the insulin receptor and the insulin-like factor-I receptor[41], the stimulatory effects of leptin on the hypothalamic-pituitary-gonadal axis[42-44], skeletal growth centers[45], and the activity of enzymes essential for the synthesis of adrenal androgens[46]. 

A number of studies have reported that children with LI are shorter than their unaffected peers[6,10-12], however, the study by Isolauri et al[12] did not adjust for the physiologic catch-up or catch-down growth that is noted in normal infants and children in the first 18 months of life as they adjust their pattern of growth away from the intrauterine environment to their genetic growth potential[47]. The studies in older children did not adjust for MPTH (genetic potential)[6,11]. Furthermore, Paganus et al[11] did not detect any association between dietary intake and nutritional status in children with cow’s milk allergy while a 21-year longitudinal Finnish study found no association between a specific genotype of LI and mean growth velocity or final mean adult height for either boys or girls[48].

Our approach to the determination of height differential between LI subjects and normal controls differed from earlier studies. Robust methodologies, such as expressing the subjects’ heights in z-score, ensured that each measurement was adjusted for age and gender. Furthermore, adjusting the height data for mid-parental height z-score ensured that genetic short stature was not a confounder in the analysis for height outcome. Finally, patients with syndromes associated short stature, for example Down syndrome, were all excluded from this study. The previous studies that investigated height differences in either LI subjects or cow’s milk avoiders did not account for all of these confounders.

This study has some limitations. First, the cross-sectional study design limited causal inference about the effects of adiposity on vitamin D or stature in LI. The relatively small sample size could have precluded the detection of subtle differences between the groups. We did not collect dietary data, such as calcium intake, on the patients as some children with LI may in fact be consuming lactose-reduced milk, milk alternatives, or dairy products with reduced lactose content. We also did not have data on parathyroid hormone levels which could be elevated in states of vitamin D deficiency and hypocalcemia. Bone age data were not available for our cohort. It is possible that adiposity could lead to bone age advancement and possibly contribute to the height differential between normal weight and overweight/obese children. However, this is less likely in a cohort of prepubertal children. 

The unique strength of this study is that it was conducted exclusively in prepubertal children. Studies in prepubertal children are a better guide to potential causal associations than studies in pubertal or postpubertal subjects because associations in childhood are less prone to confounding physiologic and lifestyle factors, such as the different stages pubertal maturation, and the effects of fluctuations in pubertal hormone levels on growth and adiposity. This prepubertal cohort represents the youngest group of subjects in whom the association between adiposity and height in LI could be demonstrated. The robust group of healthy prepubertal children in the control group ensured the validity of the anthropometric and biochemical comparisons. Furthermore, the height data were expressed in standard deviation scores, thereby adjusting for age and gender; and further adjusted for mid-parental target height to exclude the effects of genetic causes of short stature. Subjects with acquired or congenital causes of short stature were excluded from the study. 

In conclusion, we have shown that vitamin D deficiency is not a central feature of LI in prepubertal children. However, when adjusted for BMI status, both the normal weight and overweight/obese patients with LI had significantly lower 25(OH)D levels compared to the normal-weight controls. Studies examining the role of calcium on BMD in LI patients should address the vitamin D status of the cohort. This study also showed that prepubertal children with LI were not shorter than normal controls. In fact, the obese LI subjects were taller than normal weight controls, and of similar height as the overweight/obese controls. Therefore, we conclude that LI does not have a significant effect on the overall stature or vitamin D status of the affected children.

### Data depository

Our study data files are publicly deposited in the University of Massachusetts Medical School’s institutional repository, eScholarship@UMMS. The permanent link to the data is http://escholarship.umassmed.edu/datasets/1/.
